# Something Old, Something New, Something Borrowed, Something Taboo: Interaction and Creativity in Humour

**DOI:** 10.3389/fpsyg.2021.654615

**Published:** 2021-05-13

**Authors:** Vladislav Maraev, Ellen Breitholtz, Christine Howes, Staffan Larsson, Robin Cooper

**Affiliations:** Department of Philosophy, Linguistics and Theory of Science, Centre for Linguistic Theory and Studies in Probability (CLASP), University of Gothenburg, Gothenburg, Sweden

**Keywords:** humour, coronavirus pandemic, dialogue, creativity, enthymematic reasoning, interaction

## Abstract

In this paper we treat humorous situations as a series of events underpinned by topoi, principles of reasoning recognised within a socio-cultural community. We claim that humorous effect in jokes and other discourse is often created by the juxtaposition of topoi evoked. A prerequisite for this is that there is a shift where the interpreter of the discourse updates their information state with regard to a second topos being evoked. This view of humour is consistent with an incremental analysis of dialogue, and we therefore argue that interaction is central both for humour creation and interpretation. We point out some different ways in which topoi are juxtaposed in humorous dialogues as well as in jokes published in social media or in joke books, and take jokes from the coronavirus pandemic as an example because this makes lots of new topoi available and therefore offers the opportunity of creating novel jokes based on the juxtaposition of the new and existing topoi. We explore how the mechanisms of inference in dialogue can be applied to humour through the four elements from our title: old (existing), new (not previously existing), borrowed (associated with a different situation) and taboo (inappropriate in the context).

## 1. Introduction

The title of this paper is, we think, mildly humorous. We claim that the humour involves taking something known (the advice to brides to wear or carry *something old, something new, something borrowed, something blue*) and transposing it from one type of situation to another. In this case, that is from the type of situation where a wedding is taking place to the type of situation where humour is being analysed. In the process the old phrase has been slightly modified to make it fit better with the new situation type, though preserving the rhyming pattern of the original. The creation of new humour often reuses something pre-existing in this way and something about the mapping from one situation type to another creates the humorous effect. In order to study this, we take advantage of the novel situation types created by the coronavirus pandemic and examine jokes that have appeared related to it. Many, if not all, of them involve some kind of reuse in this manner. We argue that much or all of human creativity, ranging from creativity in the arts to the creation of novel sentences in everyday speech, makes use of well-known components that others will recognise and adapts them to a new situation.

A central aspect of our approach to humour is that it involves interaction. It involves an agent performing an action (linguistic or otherwise) which another agent will find funny. Normally what we call *humour* concerns actions which we intend to be experienced as funny, although of course it is possible to perform actions which are unintentionally funny. The kind of humour which is found in text, such as jokes in joke books, are special forms of this interactive process, just as literary texts are special forms of dialogue where the author is addressing the reader of the text^1^. We thus, believe that the basic notion of humour is to be revealed in the interactive process of humour which then can be recognised in such texts. Rather than studying humorous texts, as a large part of the literature on humour does (see section 2.1), we highlight the need to study the interactive process itself in order to understand the foundations of humour.

Analysing humour in terms of humorous activity (linguistic or otherwise) involving interaction between agents makes it natural to suppose that much (or perhaps all) of humour is context dependent. The mental state of the addressee also plays an important role in whether they will find it funny, including their previous knowledge and beliefs but also their tracking of the humorous action as it unfolds and the inferences that they may draw or conclusions they can surmise based on what has happened so far. Such reasoning takes time (measured in milliseconds). Once we think of humour in terms of action in this way we can begin to see why timing is such a crucial ingredient in genres like farce or stand-up comedy.

A possible objection that might be raised to this view of humour as interaction is that it is possible for a single person to find things funny without interacting with another person. We regard this in the same way as we regard talking (or thinking) to yourself. The basic strategy is interactive even if the “other” agent is the agent carrying out the original action.

In order to account for creativity in humour in this setting we need a theory of humour that is modular in the sense that we can describe a humorous exchange or a joke in terms of several elements building up an amusing situation rather than as two clashing scripts each representing different prototypes of situations. We suggest treating humorous situations as a series of events underpinned by *topoi*, principles of reasoning recognised within a socio-cultural community. Thinking of events generating humorous effects in terms of topoi rather than scripts makes possible a more fine grained analysis suitable also for humorous interactions occurring in spontaneous situations not strongly associated with particular scripts (unlike jokes). We argue that such situations, where interlocutors involved in dialogue create humorous effects by juxtaposing contrasting topoi or evoking topoi which relate in an unexpected way with the situation at hand, are the origin of the scripted situation types often drawn on in jokes.

The remainder of the paper proceeds as follows. In section 2, we motivate our dialogical approach to humour and in section 2.1 compare it to existing theories of humour. In section 2.2, we introduce the notions of *topoi* and *enthymemes* which are central to our analysis. Section 2.3 describes and motivates our main source of data: instances of humour about the 2020 coronavirus pandemic. In section 3, we present our central argument for humorous creativity by discussing each of the elements in our title—something old, something new, something borrowed, and something taboo—in turn. We put these elements together in section 4 to create our own coronavirus joke and discuss our findings and provide directions for future work in section 5.

## 2. Humorous Interaction

Many theories of humour, which we discuss further in section 2.1, focus exclusively on written versions of jokes with an idealised non-present audience. However, in reality, humour is always based in an interactive context, and, we argue, the cognitive and social mechanisms managing dialogue processes like turn taking, repair, grounding, and contextual enrichment, are also the mechanisms that allow us to produce and interpret both linguistic and non-linguistic humorous events.

The dialogicity of jokes and other humorous events is reflected in the emphasis on the sequential structure of jokes in many studies of humour (see for example Suls, 1972; Ritchie, 2018). At each increment^2^ there is a potential for participants in a humorous exchange to interpret things differently. This is often exploited in jokes. For example, the joke in (1) plays upon the fact that the perspectives of the two characters are different and this fact and the information conveyed by the joke about the specific situation is revealed to the joke hearer incrementally.

(1) from Hurley et al. (2011)
A senior citizen is driving on the highway. His wife calls him on his cellphone and in a worried voice says, “Herman, be careful! I just heard on the radio that there was a madman driving the wrong way on Route 280.” Herman says, “Not just one, there are hundreds!”

The example above illustrates dialogicity *within* a joke—the joke is set up as a dialogue between two characters with different takes on the situation. However, interaction is also fundamental in joke telling (or joke reading or joke interpretation) *events*. For example, the author of a joke book might not direct a particular joke at a specific individual. However, she must have some idealised audience in mind, one that is likely to get the joke. This means that even in contexts such as social media, humour is inherently dialogical, not just when a humorous tweet gets a response. The opportunity to respond, which may or may not be taken up, is made explicit in cases where there is a follow-up, as in example (2). A is making a joke referring to the social distancing rules introduced in the pandemic and the trope that men sometimes exaggerate their height on dating sites.

(2) from Twitter
A: guys will stand 5'8” from you and call it 6 feet
B: Most guys can't tell what six inches look like let alone 6 feet…

One important consequence of the dialogicity of humour is the possibility that interlocutors might interpret the same piece of discourse in distinct ways, just as characters within a joke can, and the source of humour is often a play on this potential for multiple interpretations. This potential is a consequence of, among other things, the inferential nature of language in use.

### 2.1. Linguistic Theories of Humour

One of the most prominent theories which emphasises the importance of linguistic understanding of humorous texts is Victor Raskin's Semantic Script Theory of Humour (Raskin, 1985). The main hypothesis of SSTH is formulated as follows:

A text can be characterised as a single-joke-carrying text if:

(a) The text is compatible fully or in part, with two different scripts;

(b) The two-scripts with which the text is compatible are opposite.

These notions of script opposition and script overlap, are often taken as the basis for “incongruity theories” of humour (e.g., Oring, 2016), which view an *incongruous* component as essential for humour. The General Theory of Verbal Humour (GTVH, Attardo and Raskin, 1991), extends the remit of SSTH, to differentiate between verbal and referential humour, and to account for the relative degree of similarity between different jokes. In GTVH, the notion of script opposition is the most abstract of the six “Knowledge Resources” which the creator of a joke may draw upon.

These, the most well-known theories of humour (SSTH, GTVH) are only concerned with humour competence (Attardo, 2010). They abstract away from the actual process of joke comprehension and do not include processing as a crucial condition for humour (Ritchie, 2018). Acknowledging Ritchie's claim about a lack of actual explanations regarding how jokes are processed as text, we view the dialogicity of joke processing as a crucial condition for getting a humorous effect that may result in amusement, a smile or laughter.

We believe that notion of scripts can be usefully cast in terms of topoi (as resources to account for different ways of opposing) and enthymemes (as arguments occurring in a dialogue or text, which evoke one or more topoi) that arise from specific interactional experiences (see section 2.2, below; Breitholtz and Maraev, 2019). We see the ability to manipulate incongruity in this way as being central to creativity in humour. Our model, which takes the dialogicity of jokes as its core insight, is also compatible with the GTVH model, providing a finer-grained way of describing the resources used in humour. We take these to be based on general resources for interaction.

In recent decades incongruity-resolution theories have become influential (Hempelmann and Attardo, 2011; Hurley et al., 2011). The key assumption is that most jokes require a *resolution* step, accounting for the decrease in oddity of the situation as a joke unfolds. However, although the notion of incongruity has been discussed for many years, it hasn't been precisely defined (though see Mazzocconi, 2019; Ginzburg et al., 2020, for recent attempts to do so), and many scholars claim that other key concepts in incongruity-resolution theories also lack precise definitions (Ritchie, 2004; Morreall, 2011; Warren and McGraw, 2016). In this work we do not aim at precisely defining incongruity, although we believe that the elements in our account can be used as the building blocks for defining (and therefore calculating) incongruity.

Ritchie (2018) emphasises the importance of explicating these so-called “theory-internal” concepts in “theory-external” terms which will arise from more general explanations relying on underlining cognitive processes, such as text comprehension. Along these lines, Attardo (2010, Chapter 3) underlines some of the necessary semantic and pragmatic tools for establishing the meanings of texts, for the purpose of accounting for humorous text beyond short jokes. We agree with the importance of explanation in terms external to humour. In our case we hope to add to this body of work by explicating our theory of humour in terms of wider notions of incremental reasoning and enthymematic inference in dialogue.

A considerable amount of research has also been done in conversational humour, mainly studying it from a qualitative and sociological perspective. For instance, Hay (2000) studies gender differences in humour production, Davies (1984) considers the group activity of “joint joking” and highlights different styles of such activity, and Günther (2003) provides conversational analysis of canned jokes and corresponding laughs in the BNC corpus. Our theory is intended to apply to these naturally occurring humorous episodes, not just to written jokes as found in joke books.

### 2.2. The Role of Inference in Humour

Jokes, like any piece of discourse that in some way involves implicit meaning, necessitate drawing on some kind of resources about the world (Yus, 2003) in order to infer from what is explicitly said. These resources could be facts, judgements about people and society, etc. which underpin inferences and associations made by interlocutors. Breitholtz (2020) discusses the link between such resources and the different types of rhetorical relations in discourse theories like Segmented Discourse Representation Theory (SDRT; Asher and Lascarides, 2003) and neo-Gricean pragmatic theories such as Relevance Theory (Wilson and Sperber, 2004).

Breitholtz and Maraev (2019) suggest analysing humorous interactions in terms of *enthymemes*, arguments where the conclusion does not follow by necessity, usually because one or more premises are not explicit in the discourse. The principles warranting enthymemes are referred to as *topoi*. Ducrot (1980, 1988) and Anscombre (1995) argue that topoi are essential not only for coherence in argumentation but for all kinds of interaction, as they supply principles of reasoning which must be recognised by an interlocutor for enthymematic discourse to make sense. For example, if Alice is going out on a rainy day, and Bob advises her to take an umbrella, it is implicit that the umbrella provides protection from the rain. If Bob in the same situation tells Alice to put on a sun hat, the comment would either not make sense to Alice, or be taken as sarcasm due to general practices associated with umbrellas and sunhats and different types of weather. Thus, it is important for understanding to base arguments on acceptable topoi.

It is important to point out that the exact nature of a topos warranting a particular argument is hard, if not impossible, to determine, as topoi are not rules of logic, but rather associative rules of thumb about how it is acceptable to reason. We know that certain pieces of discourse require underpinning by a topos to make sense, and based on intuition we could say something about some of the features present in that topos. However, sometimes it is obvious that there is more than one topos available that could successfully underpin a chain of reasoning. In our analysis it is the juxtaposition of clearly different available topoi that gives rise to a humorous effect. For example, (2) relies on two contrasting topoi: a corona-specific *safe-distance* topos that people should stay 6 feet apart and a topos, associated for example with dating apps and web sites, that men who are 5'8” tall often claim to be 6', with *6 feet* as a point of overlap between the two. We will return to this example in section 3 below.

Topoi may be very generally applicable, such as the topos that items which are not supported by anything fall to the ground, which holds in most contexts on earth. However, often topoi are specific to, or at least more strongly associated with, particular contexts. This context may be recognised by the citizens of a nation, the members of a sub-cultural group or people in a particular age span, such as children in school. Also, just as new topoi emerge when new situations arise, established topoi gradually disappear as norms and circumstances change. For example, consider the joke in (3):

(3) What game does a lady's bustle resemble?
Back-gammon! [Gammon is a type of ham.]

This joke is a word play on the name of the game backgammon and gammon as a joint of meat, the rear leg of a pig, implying that this is what a bustle^3^ looked like. This fashion of making your backside look huge was much ridiculed at the time, and there was even a particular genre of “bustle jokes.” Today, there is still an overarching topos that changing the way you look to appear more attractive is slightly ridiculous. However, this applies to things like botox, but not to dying one's hair. So, even if we know what a bustle is, the humour is less obvious to us than it would have been to a nineteenth century person who had access to a topos that if *x* uses a bustle, *x* is vain and slightly ridiculous, while no similar topos existed for example with respect to corsets.

Another example where a topos is strongly associated with a particular situation is the corona related joke in (4):

(4) “Dear Postnord Customer! The Corona pandemic poses big challenges for our company. How can we claim that we sought you, but that no-one was at home?”

The joke is a fabricated message that is written as if it comes from the Swedish postal service Postnord, which has a bad reputation for service in general. The topoi that are relevant for interpreting this joke are that since there is a pandemic, people are at home, and that Postnord tend to make excuses for not delivering, blaming the recipient, or sender for not having met the conditions for delivery.

The basic topos that this joke evolves around is the principle that if someone is at home and there is a parcel for them, the parcel can be delivered. We represent that as (a) below. In our semi-formal notation, the premises are shown above the line and the conclusion below, as is standard. The wiggly line denotes a not strictly logical chain of reasoning, as opposed to for example an if-then sequence separated by a straight line, which indicates a logical inference. These are not intended to be complete formal representations, rather as a convenient and clear way of representing our intuitions about topoi and enthymemes. More complete formal representations are shown in Breitholtz (2020). The topos that if someone is at home they can receive a delivery is acceptable to most people. It is also relatively uncontroversial that if the opposite were true, that the person who is expecting a delivery is not at home, the parcel cannot be delivered, as seen in (b).


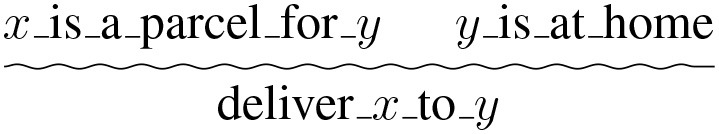
(a)


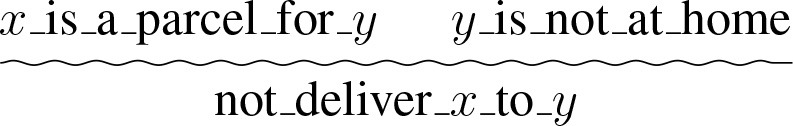
(b)

(b) licences an argument that a particular parcel hasn't been delivered by Postnord since the recipient was not at home, and is thus applicable to situations where the premises above the wiggly line in (b) are instantiated. However, there is a third topos in play here—one saying that Postnord claims (possibly falsely) that they are unable to deliver parcels since recipients are not at home.


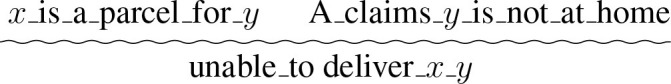
(c)

An argument based on (b) is acceptable [though possibly mistrusted due to (c)] in situations where the claim that the recipient is not at home is true or at least not clearly false. However, in times of lockdown, where the vast majority of people are at home most of the time, this is very unlikely to be the case.

### 2.3. Humour Interaction During the Coronavirus Pandemic

The 2020 coronavirus pandemic is a widely discussed global event. Such new situations introduce new concepts and beliefs into a community (in the case of coronavirus, across the globe, but in other cases in more limited groups), which quickly become shared. Examples of these concepts in the case of coronavirus include notions such as social distancing and lockdowns and more scientific concepts such as flattening the curve and R-numbers. This evolving, globally shared socio-cultural context offers a unique opportunity to explore how new humour arises from the combination of existing and new interactional resources.

The new concepts and beliefs around the coronavirus available to the community are dynamic with new concepts becoming available as resources for language users as our knowledge of the virus—and the societal changes it has brought—evolves. These can be tracked through mainstream and social media from discussions about lockdowns and stockpiling in spring 2020 to conversations about new vaccines in winter 2020 (Abd-Alrazaq et al., 2020).

Much has been written about the use of disaster humour as a psychological way to cope with uncertain and scary events, such as the explosion of the Challenger space shuttle in 1986 (Oring, 1987) and the aftermath of the 9/11 terrorist attack (James, 2015). In a similar vein there have been a few related recent studies concerned with coronavirus pandemic including a large scale psychological study of coronavirus humour perception in Italy (Bischetti et al., 2021) and a discourse analysis study of face mask memes (Dynel, 2021). However, our focus here is rather different from most studies of disaster humour, as we are interested in the interactive dynamics of humour, rather than psychological functions or motivations. We focus on the dialogue resources required to both produce new jokes or humorous utterances and how to process them.

We take our data from the coronavirus pandemic because it has led to large quantities of new information and topoi becoming widespread in society. This rapid introduction of new topoi (in this case related to the coronavirus pandemic) has led to many instances of humorous creativity in the form of jokes, memes, videos, and funny exchanges, rapidly disseminated through social media. This makes the coronavirus pandemic a perfect case study for exploring human humorous creativity, as we do in the remainder of the paper.

The examples in this paper have not been collected in a systematic way as our aim is not to provide a quantitative analysis. We rather use examples which were shared with us on social media by our own social networks—and that we found humorous—to illustrate our argument.

## 3. Elements of Humorous Creativity

In this section we discuss four elements in humour corresponding to the title of the paper:

1. Something old
2. Something new
3. Something borrowed
4. Something taboo

Our claim is not that any one of these elements is either necessary or sufficient for humour. Indeed the same utterance can be experienced as humorous by one agent and not humorous by another agent in the same situation. Furthermore, a single agent can experience something as serious at one time and then at another time find it funny (as in “We laugh about it now, but it was deadly serious at the time”). The presence of one or all of these elements is, then, no guarantee that something will be experienced as humorous.

We do, however, hypothesise that anything which is experienced as funny will have at least one, and often several, of these elements. Furthermore, it seems to us that a central aspect of creativity in humour is the reuse of something old adapted to a new situation.

In going through the four elements listed above, we will give several examples of jokes where something old and something new is combined, the “old” thereby being borrowed into a new context where it is combined with the “new.” These three (old, new, and borrowed) thus seem, at least judging from these examples, to hang together as a whole, and be directly related to topoi. Rather than attempting to isolate these factors, sections 3.1–3.3 highlight the role of the old, the new, and the borrowing, respectively. Just as in the case of our borrowed bridal saying, there is no need for these elements to be mutually exclusive, for example, a bride might borrow a brooch from her grandmother, thus fulfilling the criteria for both something “old” and something “borrowed.”

Something taboo is less central to our analysis, as not all jokes include a taboo element. However, taboos often strengthen the humour and many jokes which do not include a taboo in the strict sense of the word do relate to what Ritchie (2018) calls “impropriety.” We discuss this further in section 3.4.

### 3.1. Something Old

In this section we will look in more detail at example (5), and see how the new corona topos has been creatively combined with an already established, or “old,” topos for humorous effect. Informally, we can speak about two topoi here: the corona-specific *safe distance* topos and the pre-existing *dating website* topos, with “6 feet” as a point of overlap between the two topoi.

(5) guys will stand 5'8” from you and call it 6 feet

Information which is present in the joke needs to be integrated with pre-existing knowledge. The joke brings a few puzzles when processed, which require additional creative effort from the listener. Why do guys call the distance 6 feet when it is 5'8”? How easy is it to notice 4” difference in distance? Why does this relate to guys specifically, and not to people in general? Overall, some imagination is required from the listener.

But what can this imagination be based on? We argue that connotations of the words used play an important role, and this can be expressed in terms of the topoi that are available for conversational participants. In any given situation or context there will be several topoi which are potentially applicable, but some will be more salient than others. In the case of topoi related to the coronavirus, these are particularly salient as they are directly related to people's everyday lived experience. Much humour relies on the existence of the multiplicity of applicable topoi in any given context. More generally, jokes are often based on the asymmetry of the salience of topoi (we refer the interested reader to Breitholtz and Maraev, 2019 for discussion).

More formally we can speak of two crucial topoi; during the coronavirus pandemic people should stand 6 feet apart (to prevent the spread of the disease), which we represent as (d), and the topos that guys exaggerate their height on dating sites (e).


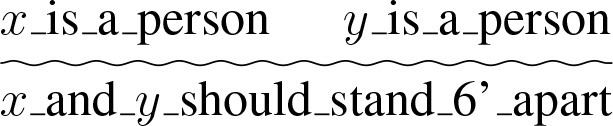
(d)


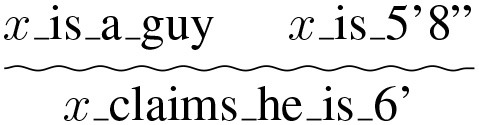
(e)

In order to see what mechanisms are required for the creative process of comprehension let us modify the joke slightly, to see which elements are required to make it comprehensible and/or humorous.

#### 3.1.1. Relocating the Joke to the UK

First, let's move our joke to the UK, where people refer to height in imperial units, but the coronavirus social distancing rule is formulated as “Stay 2 m apart from anyone not in your household”^4^. Therefore, (d) requires one or several additional premises in order to be processed. We can see (at least) two possible reasoning patterns: one option is to add the premise that person *x* and person *y* are located in the USA. Another option is to reason by seeking an analogy of the corona specific 2 m rule, that is the 6 feet rule. Overall, taking the additional premises into account, the humour ought to be less obvious (and perhaps less funny) for a Brit as compared to an American, although this can be further investigated in an empirical study.

#### 3.1.2. Relocating the Joke to Europe

In Europe, feet are not used at all in measurements. Let us try to adjust the joke to the European metrics and corona-specific rules by changing the coronavirus restriction. Given the restriction, in the joke we will need to “call it” 2 m. But what about the height? We have two alternatives: direct conversion of 4” difference (6) or picking some arbitrary height that is “not good enough” for dating (7).

(6) guys will stand 189 cm from you and call it 2 m.

(7) guys will stand 1.6 m from you and call it 2 m.

Here the corona-specific topos, similar to τ_*distancing*_ (d) but involving 2 m distance is invoked, but not the “dating website” one, because 2 m is commonly considered “too much” for a height. Therefore, the joke basically doesn't work.

The joke also does not pass the direct measurement conversion test:

(8) guys will stand 172 cm from you and call it 183 cm.

Here the coronavirus social distancing topos is no longer salient here and the “dating website” topos is not salient either. One of the possible ways to encourage associations with online dating is to substitute the very precise 183 cm by (say) 185 cm. But this would not make it humorous, just bizarre and possibly far-fetched: one can think of it as a riddle, and the solution to it is to convert cm to inches, think of it in an American context and only then get to the humour.

#### 3.1.3. Guys

One more thing to test is to break the compatibility with the old “dating website” script, or, more specifically, topos (e) which constitutes it, and is itself based on the more general topos that being tall (but not too tall, as discussed in section 3.1.2, above) is considered an attractive quality in men (at least in Western societies), such that men who do not meet the tallness criteria of attractiveness may be inclined to claim that they do in situations involving searching for a partner.

(9) people will stand 5'8” from you and call it 6 feet

Although the (USA-specific) corona social distancing topos still applies here, (9) does not invoke the same associations between height exaggeration on dating apps because “people” usually encompasses both men and women. There is no common topos about women exaggerating their height to attract a date, and different norms apply. As with 2 m for men, discussed above, 6' is generally considered excessively tall for a woman. This means that even if there were an equivalent topos about women exaggerating their height on dating apps, the heights in question would be e.g., 5'4” and 5'9”, which would not be compatible with the corona social distancing topos.

#### 3.1.4. Summary

In summary, in this section we have highlighted the role of the existing (“old”) information in the process of creating a novel joke. We have shown that to understand the humour you need to have access to the old topos (in this case the dating website topos)—which you may not if you come from Europe, where feet are not used to describe either height or distance. Additionally, you must be able to find a point of overlap between the old topos and the new topos, which may not be obvious in the case of example 2, if you only use feet for heights and not in the social distancing rules, as in the UK.

### 3.2. Something New

As previously discussed, informally we think about the creation of humorous discourse as involving something old and something new. In the case of the new jokes around the coronavirus pandemic, this means that well established and generally accepted topoi are combined in some way with topoi which are novel. We have already seen examples of this in (2) and (4).

Although the coronavirus jokes are established by combining completely new topoi with established topoi, our notion of “newness” does not rely on the acquisition of completely new topoi. In general, jokes can be created from two or more different topoi which are already available to a competent language user. What is new, we argue, is the relationship which is established between topoi, which may, for example, come from different unrelated domains, or from completely new topoi as in the coronavirus pandemic examples discussed here. As we become used to the combinations through repeated exposure, these lose their novelty and the jokes lose their humorous effect.

The novelty of a topos is not fixed, either. Repeated exposure to a topos means that the novelty value decreases and the possibility of making jokes using the topos in creative ways also diminishes. It seems likely that there is a quantifiable relationship between the novelty of topoi or combinations of topoi and how humorous they are perceived to be (as seems to be the case with so-called “Dad jokes” which may induce laughter in children who have not encountered them before, but groans from more experienced members of the language community), but this is an empirical question for future work.

The decrease of novelty of topoi is particularly clear where many new topoi quickly became shared—available as resources for a community of language users—in a short space of time, due to exceptional circumstances. In the case of the coronavirus pandemic of 2020, early on in the pandemic (before many countries went into lockdown) people started panic buying certain goods such as toilet paper. This led to the topos “if you are going to be in lockdown, you need plenty of toilet roll,” with a chain of reasoning from existing topoi that can be paraphrased as: if something is essential then you don't want to run out of it, and if you don't want to run out of something then you should buy lots of it.

Given the new premise that during a lockdown toilet roll is an essential item, and that during a lockdown there are limited opportunities for buying goods leads to a more specific version of the topos such that you should buy lots of toilet paper if you are going to be in lockdown. This led to jokes such as that in (10) when the new topoi first became shared, but these typically did not persist as the context changed and it became clear that buying toilet paper was still possible during lockdown.

(10) Why did the chicken cross the road?
She saw a shop with some toilet rolls left

In addition to the corona specific new topoi and the pre-existing old topoi, getting the joke in (10) also requires a knowledge of the joke frame in English of the classic chicken joke (11), which the lockdown chicken joke subverts and exploits.

(11) Why did the chicken cross the road?
To get to the other side

Interestingly, while the classic chicken joke is usually considered to just be absurdist^5^, subverting the notion of a chicken crossing the road for exactly the same reason a person would (which even small children can grasp), it originally may have had a double meaning relying on knowledge that where you go when you're dead can be referred to as “the other side,” which was well known when the chicken joke first appeared (presumably some time before it is first attested in print in a 1847 New York periodical), though may be a less accessible topos now [or completely unavailable, as with the “bustle” example (3)]. This additional knowledge that a (suicidal) chicken crossing a road is likely to be hit by a car and killed adds another level to our understanding of the joke^6^. This ability to get the joke at different levels is characteristic of jokes – which rely on interlocutors having different (and possibly multiple) interpretive resources available.

The dynamic nature of which topoi are salient in a particular situation also means that certain humorous comments which would not have been interpretable to us (or at least would have required a significant effort to understand) before the coronavirus pandemic now become comprehensible due to our new salient topoi, which are analogous to many from the 1918 flu pandemic, such as (12), about “flu” masks, which are also prevalent in the coronavirus pandemic (though usually referred to as “face” masks)^7^. Example (12) juxtaposes the contrasting reasons a woman might have for covering her face: either to appear alluring (as with a harem veil) or to prevent the spread of infection (in the case of the flu mask).

(12) Every woman secretly believes she would be fascinating in a harem veil. Wearing a flu mask is a good, safe way to try the effect.

Other jokes which may not be so obvious to a modern audience, such as (13) rely on the context of the 1918 flu pandemic occurring at the same time as the first World War, with the Allies fighting the Germans led by Kaiser Wilhelm II. This joke can, however, be updated to the 2020 context by simple substitution of both the disease and a controversial figure, as in (14). Whether you find this funny or not will also depend on your political persuasion, which also relies on your acceptance of a number of associated topoi.

(13) The Kaiser and the Flu are running neck and neck in the world's popularity contest.

(14) Donald Trump and the coronavirus are running neck and neck in the world's popularity contest.

### 3.3. Something Borrowed

A common technique for creating humorous effect is importing a topos (that is, *borrowing* it) from a different domain/type of situation to the context of the joke. This involves *accommodation* (Lewis, 1979; Beaver and Zeevat, 2007), integration of new information which is in some way conveyed or hinted at in an utterance but not explicitly stated. Accommodation is frequent in dialogue and often happens seamlessly as the things we accommodate are non-controversial (Larsson, 2002; Breitholtz, 2020). We believe that many or even all utterances which involve reasoning require the accommodation of topoi. Normally, although not activated before the utterance in question, this accommodated information is more or less salient in the current domain or context. However, the cases in which a humorous effect is created seem to require accommodation of topoi that are not the most salient and that need to be borrowed from a different domain or context.

In previous examples (2 and 4), we have seen how jokes rely on combining something old (in these examples, from pre-corona times) and something new (corona-related). We argue that these jokes can be described as borrowing a new topos (from the corona pandemic context) into an old context [males boasting in online dating sites and late mail delivery, respectively for (2) and (4)]. However, the borrowing effect comes out even more clearly when a new topos is borrowed into the context of a more clearly defined existing joke structure, such as knock-knock jokes. In such cases, the joke structure is assumed to be familiar to the hearer(s), and the jokes rely on jointly establishing the context of the well-known joke structure, and then breaking it by introducing a topos from the “new” context.

To make clear how these jokes rely on access to the topos to be borrowed, we will look at a dialogic exchange where a dialogue participant *lacks* sufficient knowledge of the context that the topos to be accommodated is to be borrowed from. (In this example, the borrowed topos is neither new nor corona-related, although it can be assumed that the joke was perceived as more funny when the borrowed topos was more recent and more salient than it is now.) The excerpt is an example of explicit joke telling from the British National Corpus (BNC). In this extract, 6-year old David reproduces the knock-knock joke (in line 3799) without understanding its meaning. We can say that he does not understand what is incongruous about the Avon lady knocking, which is what (allegedly!) makes the joke funny.

(15) *Phillip (46), Jane (40), Christopher (9), David (6)—at home having breakfast [BNC KCH]. Overlapping material is shown in square brackets*.

**Table:** 

David	3797	Knock, knock.
Jane	3798	Who's there?
David	3799	The Avon lady, your bell's broken!
Phillip	3800	The Avo- Avon lady?
David	3801	Mm mm.
Phillip	3802	What does she do?
	(...)	
David	3814	Dad, I don't know what an Avon lady does.
Phillip	3815	What does she do?
David	3816	I don't know.
Phillip	3817	Mm mm!
	3818	Oh!
	3819	Well she doesn't come here.
David	3820	She fixes bells.
Phillip	3821	〈laughing〉: No
David	3822	Well what [does she do?]
Jane	3823	[Guess] can't you?

**Table:** 

Christopher	3824	〈talking from other room〉 She rings the bell, she rings.
	3825	And she
Jane	3826	She co-
Christopher		〈unclear〉
Phillip	3827	Okay.
	3828	Thanks Chris.
Jane	3829	She's somebody who comes to the door and tries to sell you some make-up and perfume and toys and things.

In order to understand this joke at least two things are required: (a) knowledge of the general structure of knock-knock jokes and (b) cultural knowledge of the Avon lady being a door to door salesperson (for Avon make-up products) who, according to the longstanding advertising campaign, rings the bell (leading to the advertising slogan “Ding Dong, Avon Calling” becoming a well-known phrase^8^. This joke breaks the pattern of knock-knock jokes, as “knock-knock” doesn't generally bear any sense apart from being a set-up for an upcoming pun from the joke teller.

When Phillip asks David to explain the joke (which is not for Phillip's benefit, but because he does not expect his son to have access to the appropriate topos), David (3820) proposes a topos which is compatible with the joke (someone who fixes bells would expect a broken doorbell, and therefore knock at the door). This topos is rejected by his father, Phillip (3821), although the rejection is accompanied by laughter, which indicates an mismatch between David's topos and the actual one. David's explanation is treated by Phillip as a humorous episode, albeit an unintentional one. Later, Christopher (3824) explains what the Avon lady does, which may help David to get the joke, and Jane (3829) also adds more information which might help David to understand^9^.

Next, we will show an example of borrowing of a new (corona-related) topos into a old (pre-corona) joke context. Here, the context is again clearly identifiable (erotic role-play) although perhaps more loosely structured than the “knock-knock” joke. It is a prime example of borrowing and also highlights the temporal dynamic of dialogic jokes by invoking a so-called “garden path” mechanism (Attardo and Raskin, 1991). Ritchie (2004, 2018) calls this type of joke construction the “forced reinterpretation.” The joke teller has two possible interpretations of the joke set up in mind, or, more specifically, two topoi which can underpin the communicated enthymeme. Using the sequential ordering of the information in the joke, the joke teller boosts the saliency of one of the topoi, nudging the listener toward one of the possible interpretations. This encourages the listener to accommodate this particular topos. The punch line then subverts this accommodation, revealing another interpretation of the joke. Accommodating this second, unexpected, topos from a different domain is a case of what we call borrowing.

(16) “Darling…fancy putting on a nurse's uniform?” “Ooh, cheeky boy…you feeling horny?” “Nah…we've run out of loo roll”

A teller of the joke in (16) presents an enthymeme in the first two utterances of the joke; this enthymeme can be rephrased as “If A is persuading B into wearing a nurse's uniform, then A is feeling horny” and it is an instance of a topos similar to (f), namely that a (sexy) nurse's uniform may be worn as part of an erotic role play situation. The joke teller hints at the topos (f) using available language resources (“darling,” “cheeky boy,” etc.) making it more salient and therefore encouraging the listener into accommodating it.


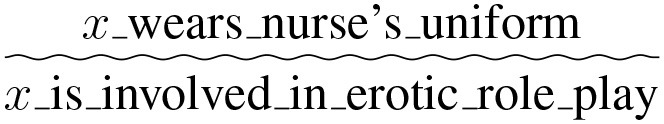
(f)

The joke teller plays on this borrowing, presenting the final utterance, which explicitly negates this assumption (“Nah…”) and providing the new reason for wearing a nurse's uniform. The reasoning behind understanding the punchline is unfolded as follows: due to the coronavirus lockdown restrictions people in general are not allowed to go out. However, these restrictions do not apply to key workers (including nurses). In the UK, for example, in the lockdown of Spring 2020, special shopping hours were introduced for NHS (National Health Service) staff, who were also exempt from quarantine restrictions. In the situation projected in the joke the reasoning is based on the lockdown specific topos that if one pretends to be a nurse, one is allowed to buy toilet paper.

In order to create a humorous effect it is not only inferences which play a crucial role, but also the *order* in which they are made. This is pointed out by Ritchie (2018, section 7.7) as a major critique against the Semantic Script Theory of Humour (SSTH) (Raskin, 1985) which claims that we can consider the text to be “joke-carrying” without sequential and procedural factors. We believe that one reason that order matters has to do with borrowing, in the sense that an established context first needs to be established so that the borrowing of a new topos creates a humorous effect, by forcing the hearer to infer and accommodate the new topos. This is an attempt to explain more specifically *why* order matters, in terms of participants' real-time inferential work on the level of topoi in dialogue.

Let's consider the following reformulation of the 5'8” joke (5) which we claim is significantly less funny:

(17) Guys keep their distance just like they lie about their height on Tinder. They will stand 5'8” from you and call it 6 feet.

Here the first sentence is the crucial inference that is assumed to be made by the listener of the joke. In our opinion, making the inferred overt ruins the humour, or at least makes the joke much less amusing. This emphasises the importance of the process of integrating new information by the listener, and the corresponding assumptions that are made by the joke teller.

Another example is given in (18), a modified version of (1). Here, we see that merely adjusting the order in which information is introduced, without making anything more explicit, seems to make a joke less funny (but perhaps more confusing).

(18) A senior citizen is driving on the highway and confronts hundreds of cars driving the wrong way. His wife calls him on his cellphone and in a worried voice says, “Herman, be careful! I just heard on the radio that there was a madman driving the wrong way on Route 280.”

To sum up, one might argue that all jokes that combine topoi from different contexts are examples of borrowing from one context into the other. However, the borrowing aspect is more clearly brought out when one context (often but not necessarily a joke-related context) is first established, and then an unexpected topos from a different context is introduced.

### 3.4. Something Taboo

Taboo subjects are those which it is not (usually) acceptable to talk about in a given society. This may be because it is repulsive (as with bodily functions, such as poo and vomit) or because it is considered morally unacceptable (such as adultery, incest, or cannibalism). Many societies have taboos about sex and death, with other taboos (for example about particular types of food) demonstrating that taboos are based on specific cultural norms.

Several of the jokes in this paper involve a taboo element. For example, in (16), the initially evoked topos is about erotic role play, which is taboo in most contexts. While we will not precisely define what is taboo we do claim that elements that are considered taboo in a particular context can create a humorous effect or enhance a joke. An example is the joke (19), below.

(19) Since everybody has now started washing their hands, the peanuts at the bar have lost their taste.

Here the communicated topoi are that the taste of people's fingers greatly contributes to the taste of communal bowls of peanuts, and if people don't wash their hands there will be traces of many things on their hands. In particular, there is a topos that people do not wash their hands after going to the toilet, so the peanuts will contain traces of urine or faecal matter—a classic taboo subject. This topos is also the basis of an urban myth claiming that there was a scientific study done on bowls of bar peanuts which found traces of a number of different urine samples^10^.

What counts as a taboo also depends on the context of the interaction (in a patient doctor interaction, for examples, bodily functions may be legitimately discussed) and is also gradient with certain topics being seen as more or less improper depending on the situation. We therefore extend the discussion in this section to cover topics which are not considered to be outright taboos, but are considered improper in some contexts.

Any element of joke can be appraised as a reference to a sensitive subject or an insult. For instance, in (5) the message (the topos) which was communicated covertly is that guys often exaggerate their height. Here the topos contains a criticism, therefore it can be considered sensitive—direct criticisms are not acceptable in some cultures—and appraised as being a laughable.

The aspect of joke impropriety is often associated with the work of Freud (1905), who distinguishes *tendenziös* (“tendentious”) elements in jokes, which refer to either hostility or obscenity, both of which directly relate to violations of social norms, including the norms of conversation. In the witty remark by Mark Twain below (20) the improper purpose of this quote is to covertly communicate the provocative idea that Wagner has no ideas. This implicit inference is achieved by contrasting the unusual topos evoked by the first sentence (that there is no law against composing music if you have no ideas) with its inverse (that Wagner composes legal music and therefore has no ideas). The humour here exploits the tendency to overextend conditionals to biconditionals, that is, if “if *a* then *b*” is true, then “if *b* then *a*” is also true, which is prevalent in human reasoning (Wason, 1968). The humour is additionally enhanced through the contradiction of the common topos that Wagner is a great composer (and great composers usually have lots of ideas).

(20) There is no law against composing music when one has no ideas whatsoever. The music of Wagner, therefore, is perfectly legal. (Mark Twain)

According to Freud, improper subjects are funny in and of themselves. However, this cannot explain the amusement caused by jokes which already establish the impropriety or taboo in the set-up. Ritchie (2018, p. 145) provides a nice example of the point of the joke being concerned not with impropriety on the general level, which was already established in the set up, but on a more precise version of the set up, revealed in the punch line. Ritchie doesn't seem to think that amusement triggered by the joke can be explained by the Freudian view: “If a topic can be mentioned in the set-up of a joke without creating humour, it is hard to see why an indirect mention should be the cause of amusement.” (Ritchie, 2018, p. 145).

(21) recited by Ritchie (2018) from Tibballs (2000) A woman was in bed with her husband's best friend when the phone rang. After hanging up, she turned to her lover and said: “That was Jim, but don't worry, he won't be home for a while. He's playing cards with you.”

We agree that if you analyse the improper content—the adulterous liaison—on a general level, it should not be more amusing in the punchline than in the set-up. However, our approach provides greater granularity based on which topoi are available at different points in comprehension of the joke: (i) the setup invokes the improper topos of the adulterous wife, and (ii) the punchline invokes another improper and contrasting topos of adulterous husband through employing additional inference mechanisms enlisted in the previous sections.

## 4. Creating a Joke

Now let's use the elements described above to be creative and come up with our own (mildly) humorous offering.

(22) My gran's got coronavirus. I'm not worried though—she's been 35 since 1970

Here, the *something new* is the coronavirus topos that old people are more at risk of severe illness or death from coronavirus, and the novel juxtaposition of this topos with existing topoi. *Something old* includes the lexical associations from “gran”—namely that a person described as gran is female and old (we also believe such lexical aspects can be described using topoi—see e.g., Breitholtz and Howes, 2020, with features usually considered to be part of a word's meaning also being defeasible inferences—for example, it is not a necessary condition of a gran that they are old). The common pre-existing topos that is here *something borrowed* is that older women sometimes lie about their age because youth is considered an attractive quality in women (analogously to men exaggerating their height in our earlier example). *Something taboo* is the inference about death and the joke teller apparently being indifferent to the possibility of their gran dying (before we get to the punchline). It is also considered improper to do (supposedly covert) things to make yourself appear more attractive—such as lie about your age for 50 years.

## 5. Discussion

This paper takes an interactive perspective on humour and humorous creativity. We have suggested that humour can be analysed using the resources and theoretical frameworks developed for more general studies of dialogue and interaction. We have taken a closer look at some key elements at play and shown how these arise from the dialogue context in which jokes and other types of humour occur. We have argued that inference plays an important part in humour and that this inference can be analysed in terms of a notion of topos closely related to Aristotle's notion. We have also argued that creativity in humour involves more than simply saying something new, but rather lies in the combination of something new with something old which is recognised by the addressee. Much of humour seems to rely on borrowing a topos from one domain and inserting it into a new domain. Referring to something taboo can add humorous spice to the mix. What we do not claim to have done is provide a complete story about what it is about particular instances of humour which makes them humorous in a particular context, as opposed to merely miscommunication or metaphor.

In this work we also were intentionally agnostic about the notions of incongruity and clash. These are common notions in theories of humour, with hitherto unexplored parallels in dialogue research [for example in research on conversational repair (Hayashi et al., 2013) and prediction error (Garrod and Pickering, 2013)]. In future work we plan to explore these parallels.

Describing and explaining jokes on the level of topoi allows fine-grained manipulation of jokes, and thereby makes it possible to evaluate theories empirically and experimentally. In this paper, we suggested that many jokes involve combining old and new topoi, often borrowing new topoi into an established context, and often evoking taboo or improper topoi. We tested these assumptions informally here, manipulating some of these aspects by replacing specific topoi to generate new variants of existing jokes, and subjectively assessing their funniness, as in Example 2 with variants in Examples 6–8. Similarly, we showed how the temporal aspect of jokes highlighted by the notion of borrowing can be crucial to a joke, as in the variant in Example 18 of the joke in Example 1.

A natural progression of this work is to account for humour in a more precise way, following the work of Breitholtz and Maraev (2019) who use Type Theory with Records (Cooper, 2005) to provide a formal representation of how a particular joke plays out. A general formal model of humorous interaction could, among other things, provide a more precise definition of incongruity in humour, taking inspiration from incongruity related to laughter as discussed in Ginzburg et al. (2015, 2020). Such a model could be tested and evaluated and potentially also feed into research on artificial intelligence (AI) allowing conversational AI to understand and generate creatively humorous contributions (Maraev et al., 2020).

Creativity in humour is, we have suggested, not the creation of something entirely new, but rather a novel recombination of existing resources. In this way it is similar to creativity in the arts. For example, creativity in music is often perceived as a clever modification of an existing musical language such as a slight change to an existing harmonic progression or a bringing together of distinct musical resources, e.g., importing features of jazz or gamelan into western art music (Cooper, 2013). If the connections that are being made to existing resources are not recognised by the audience, then the art work is not perceived as creative or even as art. The case of creativity in humour is essentially similar. An attempted joke which the audience cannot connect to anything they previously knew in the ways that we have suggested will be at best perceived as strange or incoherent, but not funny.

## Data Availability Statement

The original contributions presented in the study are included in the article/supplementary material, further inquiries can be directed to the corresponding author/s.

## Author Contributions

All authors contributed to the discussion and research of examples. VM, EB, and CH wrote the paper with contributions from SL and RC.

## Conflict of Interest

The authors declare that the research was conducted in the absence of any commercial or financial relationships that could be construed as a potential conflict of interest.

## Footnotes

^1^It has been claimed that text in general is dialogical in nature (e.g., Voloshinov and Bachtin, 1986). We do not take a stand on this here, but argue only the weaker position that at least written jokes have a dialogical and interactive nature.

^2^In dialogue research, what is considered an increment is not necessarily a complete sentence or utterance but can be—and often is—a smaller phrasal unit, or even a word (Howes and Eshghi, 2017). We adopt this more finely grained notion of incrementality here.

^3^A type of woman's undergarment used in the mid to late nineteenth century that added volume to the back part of the skirt just below the waist.

^4^https://www.gov.uk/guidance/national-lockdown-stay-at-home.

^5^Wikipedia, for example, describes it as anti-humour, https://en.wikipedia.org/wiki/Why_did_the_chicken_cross_the_road%3F.

^6^See e.g., https://www.esquire.com/uk/life/news/a12346/the-upsetting-true-meaning-of-that-why-did-the-chicken-cross-the-road-joke/.

^7^Taken from https://www.smithsonianmag.com/history/memes-1918-pandemic-180975452/.

^8^See e.g., https://www.youtube.com/watch?v=66IWgU9AAis from 1956.

^9^Note that understanding a joke and finding it funny are not the same thing. We do not go into this distinction here.

^10^https://www.cottagesmallholder.com/peanuts-or-peenuts-2054/.
